# Aloperine Protects Mice against DSS-Induced Colitis by PP2A-Mediated PI3K/Akt/mTOR Signaling Suppression

**DOI:** 10.1155/2017/5706152

**Published:** 2017-09-19

**Authors:** Xiaoxia Fu, Fei Sun, Faxi Wang, Junai Zhang, Biying Zheng, Jixin Zhong, Tiantian Yue, Xuebao Zheng, Jun-Fa Xu, Cong-Yi Wang

**Affiliations:** ^1^Department of Clinical Immunology, Institute of Laboratory Medicine, Guangdong Medical University, No. 1 Xincheng Road, Dongguan 523808, China; ^2^The Center for Biomedical Research, Key Laboratory of Organ Transplantation, Ministry of Education, Tongji Hospital, Huazhong University of Science and Technology, Wuhan 430030, China; ^3^Guangdong Provincial Key Laboratory of Medical Molecular Diagnostics, No. 1 Xincheng Road, Dongguan 523808, China; ^4^Cardiovascular Research Institute, Case Western Reserve University, 2103 Cornell Road, Cleveland, OH 44106, USA; ^5^Mathematical Engineering Academy of Chinese Medicine, Guangzhou University of Chinese Medicine, Guangzhou 510006, China

## Abstract

Colitis is a major form of inflammatory bowel disease which involved mucosal immune dysfunction. Aloperine is an alkaloid isolated from the shrub *Sophora alopecuroides* L. and has been recognized as an effective treatment for inflammatory and allergic diseases. The present study aimed to examine the molecular mechanisms underlying aloperine-mediated colitis protection. We found that aloperine treatment improved colitis induced by dextran sodium sulfate (DSS) based on body weight, disease activity index, colonic length, and spleen index. Aloperine also effectively attenuated DSS-induced intestinal inflammation based on the pathological score and myeloperoxidase expression and activity in colon tissues. In addition, aloperine regulated T-cell proportions and promoted Foxp3 expression in the spleens and mesenteric lymph nodes of DSS-induced colitis mice and in the spleens of the *Foxp3*^GFP^ mice. Aloperine inhibited Jurkat and mouse naïve T-cell apoptosis. Furthermore, aloperine inhibited PI3K/Akt/mTOR signaling and upregulated PP2A expression in the DSS-induced colitis mice and in Jurkat cells, but LB-100 (PP2A inhibitor) resulted in an elevated Akt activity in Jurkat cells, activated T-cells, and human splenic mononuclear cells. Aloperine inhibited T-cell and lymphocyte proliferation, but LB-100 reverse these effects. In conclusion, aloperine regulates inflammatory responses in colitis by inhibiting the PI3K/Akt/mTOR signaling in a PP2A-dependent manner.

## 1. Introduction

Inflammatory bowel disease (IBD) is a familial spontaneous autoimmune disease, but its pathogenesis remains enigmatic. The highest rates of incidence and prevalence of ulcerative colitis (UC) and Crohn's disease (CD) have been reported in Europe and the USA, and these rates have recently increased 24-fold in China [1]. Moreover, none of the existing drugs are adequately efficient to provide a prominent relief to the patients with IBD [2]. Mucosal immune dysfunction, especially the overexpression of T-cells, plays a major role in IBD pathogenesis [3]. Regulatory T-cells (Tregs) and T helper cell 17 (Th17) are the most abundant CD4^+^ T-cell subsets in the lamina propria during the steady state [4, 5]. Th17 cells may enhance intestinal inflammation, whereas Tregs might inhibit the inflammatory responses [6]. The differentiation of Tregs is modulated by the expression of the lineage-specific transcription factor forkhead box P3 (Foxp3), which can suppress autoimmune diseases in normal individuals. Tregs regulate the immune system by secreting suppressive cytokines such as tumor growth factor- (TGF-) *β* and interleukin- (IL-) 10 [7]. PI3K/Akt/mTOR signaling negatively modulates Treg functions and is involved in several diseases including cancer, ischemic disease, and inflammation [8, 9]. An effective inhibitor of the PI3K/Akt/mTOR pathway might contribute to the treatment of IBD.

Previous studies have shown that aloperine downregulates PI3K/Akt/mTOR signaling in HCT116 human colon cancer cells [10]. Our group confirmed that aloperine significantly attenuated ionizing radiation- (IR-) induced PI3K activation [11]. Aloperine is an alkaloid isolated from the shrub *Sophora alopecuroides* L. and has been recognized as an effective treatment for inflammatory and allergic diseases [11–17]. It has been shown to increase CD4^+^CD25^+^ cells and IL-10 levels as well as alleviate inflammation in mice with 2,4,6-trinitrobenzenesulfonic acid- (TNBS-) induced colitis [16].

Protein phosphatase 2A (PP2A) is a multimeric enzyme that contains a scaffolding A subunit, a regulatory B subunit, and a catalytic C subunit [18]. Akt is one of the major substrates of PP2A, which phosphorylates Akt at serine 473. Inhibition of PP2A results in an elevated Akt activity [19]. PP2A has been identified as a key regulator of PI3K/Akt activation and is involved in the development of tumors and autoimmune diseases [20, 21].

Dextran sodium sulfate- (DSS-) induced murine colitis is regarded as a classical model for intestinal inflammation [22]. The aim of the present study was to examine the molecular mechanisms underlying aloperine-mediated colitis protection. This could provide novel insights into the mechanisms of IBD and improve the treatment strategies.

## 2. Materials and Methods

### 2.1. Animals

C57BL/6 mice (10–12 weeks old, equal number of males and females) were purchased from the Hubei Provincial Center for Disease Control and Prevention (Wuhan, China). Twelve-week-old male *Foxp3*^GFP^ mice were obtained from Jackson Laboratory (Bar Harbor, ME, USA). The mice were housed at the Center for Experimental Animals, Tongji Hospital, Huazhong University of Science and Technology, for at least 1 week before any experiment. All experiments were approved by the Institutional Animal Care and Use Committee of Tongji Hospital, Huazhong University of Science and Technology (2015 IACUC: number 595).

### 2.2. DSS-Induced Colitis and Aloperine Treatment

Experimental colitis was induced by the administration of 3% DSS (molecular weight 3600–5000 kDa, MP Biomedicals, San Francisco, CA, USA) for 7 days, as described previously [23]. Aloperine (purity ≥ 98%, Kmaels, Shanghai, China; catalog number Q1962) was solubilized in saline containing 5% acetic acid [12]. Seventy C57BL/6 mice were randomly divided into five groups (14 mice/group) (Supplementary Figure 1 available online at https://doi.org/10.1155/2017/5706152): (A) negative control (control) group: the mice were intragastrically administered an equivalent volume of 5% acetic acid in saline and provided distilled drinking water for 14 consecutive days; (B) aloperine group (Alo): the mice were intragastrically administered aloperine solution at a dose of 40 mg/kg/day, according to body weight, for 7 consecutive days and simultaneously provided distilled drinking water; (C) aloperine-DSS group (Alo + DSS): the mice were administered aloperine solution for 7 consecutive days with DSS in the drinking water; (D) DSS-aloperine group (DSS + Alo): the mice were provided DSS in the drinking water for the first week and then intragastrically administered aloperine solution during the second week; and (E) DSS group (DSS): the mice were intragastrically administered 5% acetic acid in normal saline for 14 consecutive days and provided DSS in the drinking water for the first week and distilled drinking water during the second week. Subsequently, the mice were sacrificed with sodium pentobarbital on day 14. Serum, spleen, colon, and mesenteric lymph nodes (MLNs) were harvested.

Twelve *Foxp3*^GFP^ mice were randomly divided into two groups (6 mice/group): (A) Alo group: the mice were intraperitoneally injected with aloperine solution (20 mg/kg body weight) as a volume of 0.1 mL/10 g of body weight once per day for 3 consecutive days, and (B) negative control group: the mice were intraperitoneally injected with an equivalent volume of 5% acetic acid in normal saline. The mice were sacrificed by spinal dislocation on day 4.

### 2.3. Disease Activity Index (DAI) and Spleen Index (SI)

Each mouse was observed daily, and the DAI was assessed based on the clinical manifestations of diarrhea, bloody stool, and weight loss [24]. The stool consistency was checked for diarrhea, and the feces were screened for blood using a fecal occult blood kit (ABON Biopharm Co. Ltd., Hangzhou, China). The SI was calculated as the ratio of the spleen weight to the body weight on day 14 when the animals were euthanized [25].

### 2.4. Histopathological Score and Immunohistochemistry (IHC)

The colon tissues were stained with hematoxylin and eosin (H&E). The degree of colon inflammation was analyzed according to a previously described scoring system [26]. Myeloperoxidase (MPO) was detected by established IHC techniques [11].

### 2.5. Measurement of MPO Activity

The MPO activity in the colon or serum was measured using an MPO activity testing kit (Nanjing Jiancheng Bioengineering Institute, Nanjing, China).

### 2.6. Western Blotting Analysis and Real-Time PCR Reactions

The real-time PCR method has been described in a previous paper [27]. The primers are presented in Table 1. The cells or mouse colon tissues were lysed for protein immunoblotting, as described previously [28]. The membrane was probed with antibodies (1 : 1000 dilution) including PI3K p85, p-PI3K p85, Akt, p-Akt, mTOR, p-mTOR, Bcl-2, Mcl-1, Bax (Cell Signaling Technology, Danvers, MA, USA), PHLPP1, PML, MPO, p-IKB*α*, *β*-actin, cleaved caspase-3, Foxp3 (Santa Cruz Biotechnology, Santa Cruz, CA, USA), PP2Aa, PP2Ab, and PP2Ac (Protech, Wuhan, China). The intensity of each band was analyzed using the densitometry feature in the ImageJ software (http://rsb.info.nih.gov/ij/).

### 2.7. Flow Cytometry

Lymphocytes were isolated from the C57BL/6 mouse spleens and MLNs in single-cell suspensions. The cells were stained with fluorescence-labeled antibodies against CD4-FITC and CD44-APC at 4°C for 30 min. The samples were stained with antibodies against Foxp3-PE (NRRF30, eBioscience Inc., San Diego, CA, USA) at room temperature for 30 min in the dark after using a Foxp3 fixation/permeabilization kit (BD Biosciences, San Diego, CA, USA) [29]. The lymphocytes collected from the *Foxp3*^GFP^ mouse spleens were stained with fluorescence-labeled antibodies against CD4-PE and CD62L-APC at 4°C for 30 min. Flow cytometry was performed on an LSRII cytometer (BD Biosciences) and analyzed with the FlowJo 7.6 software. Anti-mouse CD4-FITC (GK1.5) and Foxp3-PE (NRRF30) were purchased from eBioscience. CD62L-APC (MEL-14) was purchased from BD Biosciences. CD4-PE (GK1.5) and CD44-APC (IM7) were obtained from BioLegend Inc. (San Diego, CA, USA).

### 2.8. Preparation of Jurkat Cells for Confocal Imaging

Jurkat cells, an acute T lymphoblastic leukemia cell line, were purchased from Clontech Laboratories Inc. (CA, USA). The cells were cultured with 0.5 mM aloperine for 48 h, followed by staining with a primary antibody against PP2Ab (rabbit, 1 : 100) [11].

### 2.9. Mouse Naïve CD4^+^ T-Cell Isolation and Differentiation

One day before T-cell isolation, culture plates were coated with 5 *μ*g/mL of anti-CD3 and 1 *μ*g/mL of anti-CD28 antibodies in sterile PBS and incubated overnight at 4°C. Lymph nodes and spleens were isolated from 8-week*-*old C57BL/6 female mice using a mouse naïve T-cell isolation kit (STEMCELL Technologies EasySep™, catalog number 19782, CA, USA). After isolation, the naïve T-cells were seeded into the wells with 20 ng/mL IL-2 for stimulation and collected after 24 h to assess the activation. The remaining cells were stimulated with 20 ng/mL IL-2 and 5 ng/mL TGF-*β* and collected after 72 h to test their differentiation. Anti-CD3, anti-CD28, IL-2, and TGF-*β* were purchased from R&D Systems (MN, USA). The Mouse Treg Isolation Kit (STEMCELL Technologies EasySep, catalog number 19852) was used to isolate mouse Tregs.

### 2.10. Isolation of Spleen Mononuclear Cells

Spleen mononuclear cells were isolated from C57BL/6 mice or healthy volunteers by density gradient centrifugation using a mouse or human lymphocyte separation medium (Dakewe, Shenzhen, China), respectively. Culture plates were coated with 20 ng/mL IL-2, 5 *μ*g/mL anti-CD3, and 1 *μ*g/mL anti-CD28 in sterile PBS overnight at 4°C. On the following day, spleen mononuclear cells were isolated and seeded into the wells for 24 h. Human splenic cells were isolated from healthy donors at the Institute of Organ Transplantation, Tongji Hospital (TJ-IRB20160601).

### 2.11. Mixed Lymphocyte Reaction

Splenocytes (1 × 10^5^ cells/well) isolated from one C57BL/6 mouse and one BALB/c mouse were mixed and cultured in a 96-well round-bottom microculture plate (Corning Inc., Corning, NY, USA). The splenocytes from the BALB/c mouse were used as allogeneic stimulators and prepared by pretreating the cells (1 × 10^7^ cells/mL) with 50 *μ*g/mL of mitomycin C (Hisun Pharmaceutical Co. Ltd., Hangzhou, China) at 37°C for 30 min. Splenocytes from the C57BL/6 mouse were cultured without stimulatory cells and used as the negative control. Mixed cell cultures treated with 2 *μ*g/mL concanavalin A (ConA) (Sigma, USA) were used as the positive control. The cultures were maintained for 5 days. The cell viability was estimated quantitatively using a Cell Counting Kit-8 assay as described below.

### 2.12. Cytotoxicity Assay

The cytotoxicity assay was conducted using a Cell Counting Kit-8 (CCK-8; Dojindo, Kumamoto, Japan). The cells were seeded in 96-well plates at a density of 3000 cells/well and cultured with the indicated treatment.

### 2.13. Cell Culture and Aloperine Treatment

Jurkat cells, mouse naïve T-cells, mouse Tregs, and human spleen mononuclear cells were cultured in RPMI 1640 medium supplemented with 10% fetal bovine serum (Gibco, Thermo Fisher Biotechnology, Waltham, MA, USA). Aloperine was solubilized in saline containing 0.08% acetic acid.

### 2.14. Inhibitors

LB-100 (PP2A inhibitor), wortmannin (PI3K inhibitor), and perifosine (p-Akt inhibitor) were obtained from Selleck Chemicals (Houston, TX, USA).

### 2.15. Statistical Analysis

Data were analyzed using the GraphPad Prism 5.0 software and presented as the mean ± standard error of mean (SEM). Data were analyzed by one-way analysis of variance (ANOVA) followed by the Tukey post hoc test or independent sample *t*-test, as appropriate. In all statistical analyses, *P* value < 0.05 was considered statistically significant.

## 3. Results

### 3.1. Alo Treatment Ameliorates the General Symptoms of Murine Colitis

**Figure 1:**
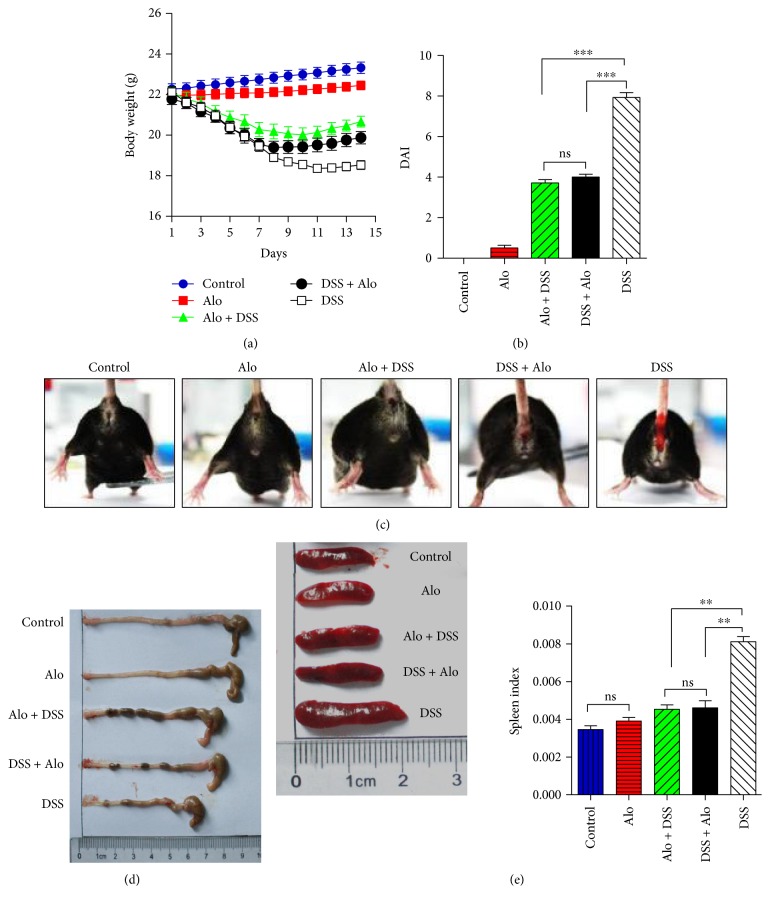
Aloperine treatment improves colitis induced by dextran sodium sulfate (DSS). (a) Body weight changes in the five groups over a 14-day period. (b) The disease activity index (DAI) in the five groups of mice on day 7. (c) Representative images showing the stool conditions on day 7. (d) Representative images showing the colon lengths on day 14. (e) Representative images of the spleen and spleen index (SI) on day 14. The data represent the mean ± standard error of mean (SEM) of 14 mice analyzed per group. ^∗∗^*P* < 0.01. ^∗∗∗^*P* < 0.001. ns: not significant.

The aloperine solution was administered orally in C57BL/6 mice with DSS-induced acute colitis. On day 7, we observed that the mouse stools were mostly normal in both the control and Alo groups (Figure 1(c)). The classical manifestations of colitis appeared in all the mice in the DSS group, including the most severe liquid or bloody stool, weight loss, and high disease activity index (DAI). In the DSS + Alo and Alo + DSS groups, the body weight first decreased, but took an increasing trend by day 8, and remained higher than that in the DSS group (Figure 1(a)). The DAI in the Alo + DSS and DSS + Alo groups was similar but significantly lower than that in the DSS group (Figure 1(b)).

After 2 weeks, all the mice were euthanatized. The colon length was shortest in the DSS group, indicating severe destruction, while the colon length in the Alo + DSS and DSS + Alo groups was longer (Figure 1(d)). The SI is related to the immunosuppressive functions. All mice in the DSS group developed splenomegaly, but those in the Alo + DSS and DSS + Alo groups were healthier (Figure 1(e)), as shown by lower SI in the Alo + DSS and DSS + Alo groups compared with the DSS group; there were no differences in SI among all other groups.

### 3.2. Aloperine Alleviates Inflammation and Reduces MPO Activity in Murine Colitis

**Figure 2:**
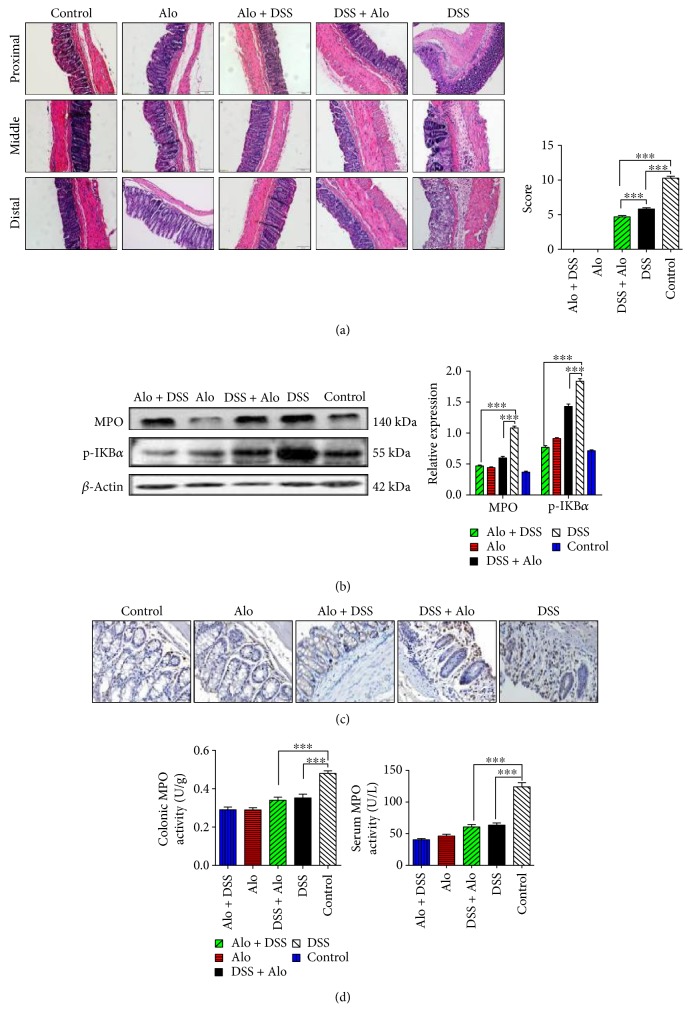
Aloperine inhibits inflammatory cell infiltration in the colon in DSS-induced colitis mouse model. (a) Histological analysis of the severity of three colon sections in mice. Left panel: the proximal, middle, and distal colons were examined using hematoxylin & eosin (H&E) staining (magnification: 20x, scale bar: 50 *μ*m). Right panel: the histological scores were graded and presented as the mean ± SEM of 14 mice analyzed per group. (b) Western blotting analysis of myeloperoxidase (MPO) and p-IKB*α* expression in each group. (c) Immunohistochemistry (IHC) analysis of MPO expression in each group (magnification: 40x). (d) Serum (right) and colonic (left) MPO activity indices in each group. The data are presented as the mean ± SEM of six mice analyzed per group (b, c, and d). ^∗∗∗^*P* < 0.001.

The DSS group exhibited severe inflammatory responses, including intense infiltration of inflammatory cells into the lamina propria, mucosal edema and erosion, ulcer and crypt distortion, and destroyed placenta percreta. The acute inflammation of the intestinal tracts in both treatment groups was greatly ameliorated, but the effect was slightly greater in the Alo + DSS group (Figure 2(a)). These data suggested that treatment with aloperine might improve murine colitis.

Western blot indicated that DSS increased MPO and p-IKB*α* protein levels, two specific markers for assessing inflammation, while aloperine inhibited MPO and p-IKB*α* protein levels (Figure 2(b)). Aloperine also alleviated the interstitial infiltration of neutrophils indicated by MPO expression by IHC staining (Figure 2(c)). In addition, DSS increased the serum and colonic MPO activity, while aloperine suppressed the serum and colonic MPO activity in murine colitis (Figure 2(d)). Thus, we concluded that aloperine effectively attenuated DSS-induced intestinal inflammation.

### 3.3. Aloperine Regulates the Proportion of Tregs

**Figure 3:**
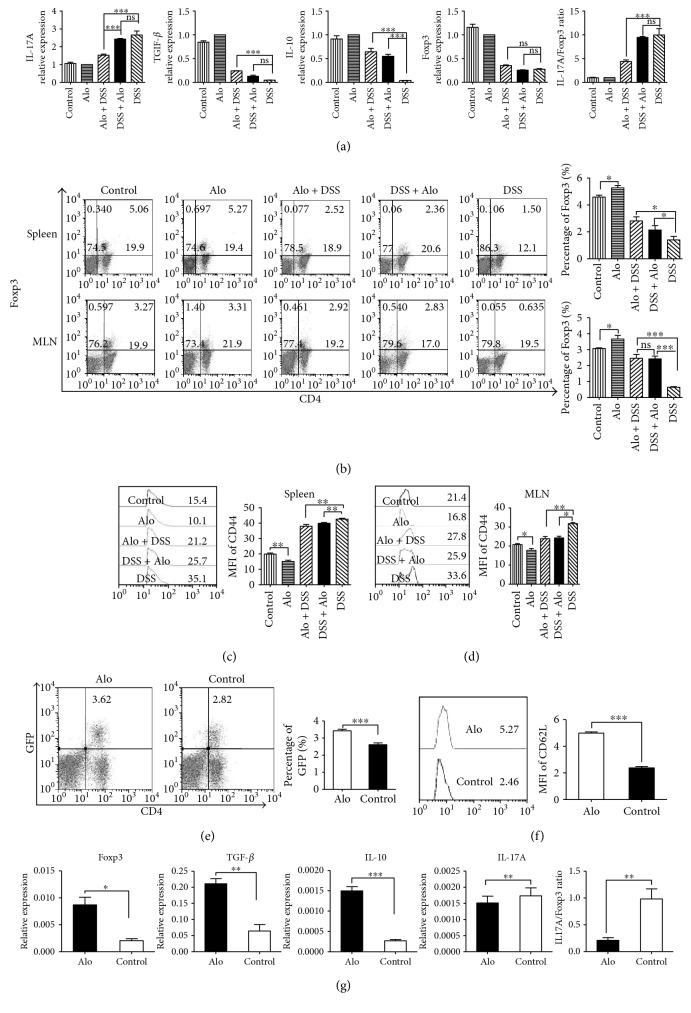
Aloperine regulates T-cell proportions in spleens and mesenteric lymph nodes (MLNs) of DSS-induced colitis mouse model and in the spleens of the *Foxp3*^GFP^ mice. (a) Representative interleukin- (IL-) 17A, tumor growth factor- (TGF-) *β*, IL-10, forkhead box P3 (Foxp3) mRNA expressions, and IL-17A/Foxp3 ratio in the colon in each group. (b) Representative CD4^+^Foxp3^+^ T-cell frequencies in the spleens and MLNs in each group. (c, d) Mean fluorescence intensity (MFI) of CD44 in the spleens and MLNs in each group. (e) Representative CD4^+^Foxp3^+^ T-cell percentages in the spleens of the *Foxp3*^GFP^ mice. (f) MFI of CD62L in the spleens of the *Foxp3*^GFP^ mice. (g) Real-time PCR analysis of *IL-17A*, *TGF-β*, *IL-10*, *Foxp3* mRNA expression, and *IL-17A*/*Foxp3* ratio in mouse Tregs after a 24 h treatment with aloperine (0.25 mM). The data are presented as the mean ± SEM of 6 mice analyzed per group. ^∗^*P* < 0.05, ^∗∗^*P* < 0.01, and ^∗∗∗^*P* < 0.001. ns: not significant.

DSS increased the levels of *IL-17A* mRNA expression, decreased *TGF-β*, *IL-10*, and *Foxp3* mRNA expressions, and increased the *IL-17A*/*Foxp3* ratio. *IL-17A* mRNA expression was decreased after aloperine treatment, especially in the Alo + DSS group. The *IL-17A*/*Foxp3* ratio was found to be lower in the Alo + DSS group than in the DSS group, but there was no difference between the DSS and DSS + Alo groups. The mRNA expressions of the anti-inflammatory cytokines *TGF-β* (Alo + DSS group only) and *IL-10* (Alo + DSS and DSS + Alo groups) were increased, while the expression of *Foxp3* did not change, compared with the DSS group (Figure 3(a)).

DSS decreased the frequency of CD4^+^Foxp3^+^ T-cells in the spleens and MLNs. Under inflammatory conditions, higher frequencies of CD4^+^Foxp3^+^ T-cells in the spleens and MLNs in the Alo + DSS and DSS + Alo groups were observed in the presence of aloperine compared to the DSS group (Figure 3(b)).

Moreover, DSS increased the frequency of CD44^+^CD4^+^ T-cells in the spleens and MLNs, while we detected a lower frequency of CD44^+^CD4^+^ T-cells in the spleens and MLNs of the mice treated with aloperine (Figures 3(c) and 3(d)). Furthermore, we found higher levels of Foxp3 and CD62L in the *Foxp3*^GFP^ mice intraperitoneally injected with aloperine for 3 days compared to the control group under normal physiological conditions (Figures 3(e) and 3(f)). Real-time PCR showed that aloperine promoted the expression of *Foxp3* and the anti-inflammatory cytokines *IL-10* and *TGF-β* in mouse Tregs. In contrast, the proinflammatory cytokines *IL-17A* and *IL17A*/*Foxp3* ratio were decreased (Figure 3(g)).

Therefore, we concluded that aloperine alleviated the inflammation in mouse colon and regulated the immune system homeostasis. In addition, aloperine might promote Foxp3^+^ Tregs and reduce T-cell activation independently from the administration route (intragastric or intraperitoneal).

### 3.4. Aloperine Inhibits T-Cell Apoptosis

**Figure 4:**
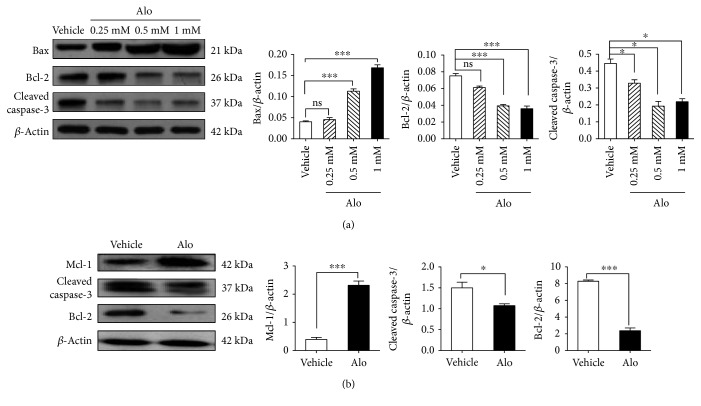
Aloperine inhibits apoptosis in T-cells. (a) Western blotting was used to assess the expressions of cell apoptosis-related proteins (Bax, Bcl-2, and cleaved caspase-3) in Jurkat cell treatment with aloperine (0, 0.25, 0.5, and 1 mM) for 24 h. (b) Western blotting analysis of Mcl-1, Bcl-2, and cleaved caspase-3 expressions in mouse naïve T-cells following 24 h of treatment with aloperine (0.25 mM). The data are shown as the mean ± SEM from three independent experiments. ^∗^*P* < 0.05 and ^∗∗∗^*P* < 0.001. ns: not significant.

Studies have shown that moderate concentrations of aloperine are effective against tumor cells and safe for normal cell viability [10, 15]. Treatment with aloperine for 24 h enhanced the expression of Bax (0.5 and 1.0 mM) but decreased the expression of Bcl-2 (0.5 and 1.0 mM) and caspase-3 (0.25, 0.5, and 1.0 mM) in Jurkat cells (Figure 4(a)). In addition, we observed that aloperine decreased the Bcl-2 and cleaved caspase-3 levels but raised the expression of Mcl-1 in mouse naïve T-cells (Figure 4(b)).

### 3.5. Aloperine Suppresses PI3K/Akt/mTOR Signaling In Vitro and In Vivo

**Figure 5:**
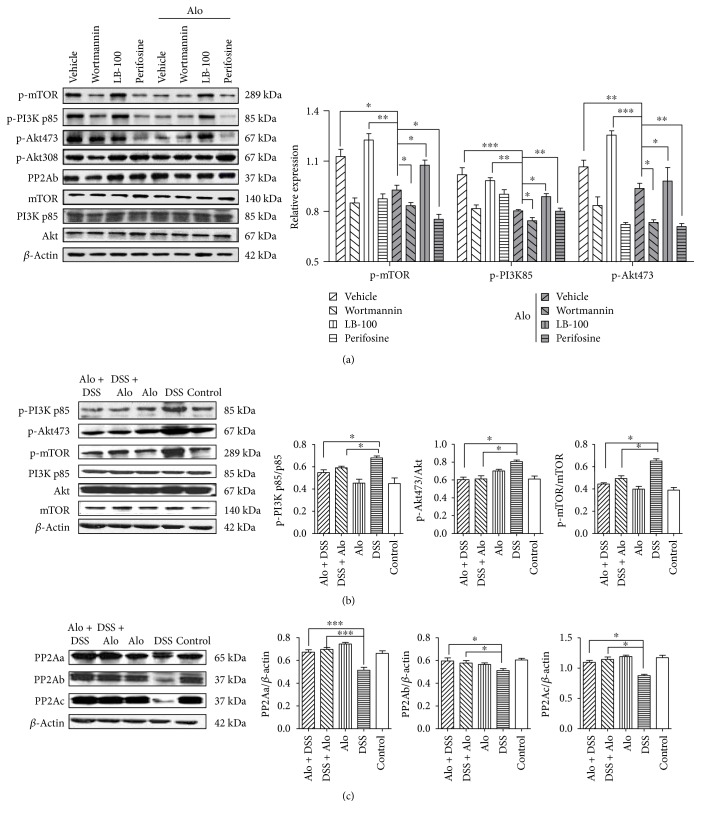
Aloperine regulates PI3K/Akt/mTOR signaling *in vitro* and *in vivo*. (a) Western blotting analysis of PI3K/Akt/mTOR in Jurkat cells after 24 h treatment with 0.5 mM aloperine with or without different inhibitors (10 nM wortmannin (PI3K inhibitor), 4 *μ*M LB-100 (PP2A inhibitor), or 4 *μ*M perifosine (p-Akt inhibitor)). The data are shown as the mean ± SEM from three independent experiments. (b) Western blotting analysis of PI3K/Akt/mTOR in the mouse colon. (c) Western blotting analysis of protein phosphatase 2A (PP2A) in the mouse colon. The data are shown as the mean ± SEM of 6 mice analyzed for each group. ^∗^*P* < 0.05, ^∗∗^*P* < 0.01, and ^∗∗∗^*P* < 0.001.

Western blotting showed that different aloperine concentrations suppressed the activation of PI3K/Akt/mTOR signaling in Jurkat cells (Supplementary Figure 2A). We cultured the Jurkat cells with aloperine (0.5 mM) alone or in the presence of the PI3K inhibitor wortmannin (10 nM) and the p-Akt inhibitor perifosine (4 *μ*M) for 24 h. This treatment of the Jurkat cells with aloperine in the presence of inhibitors resulted in an enhanced suppressive effect on mTOR, PI3K, and Akt compared to the treatment with aloperine alone (Figure 5(a)). In agreement with this result, significantly lower expressions of the downstream molecules, PI3K p85, Akt, and mTOR, were detected in the colons of mice from the two treatment groups compared to the DSS group (Figure 5(b)).

### 3.6. PP2A Is a Vital Molecule Mediating the Aloperine-Induced Suppression of PI3K/Akt/mTOR Signaling

Western blotting showed that 1 mM aloperine increased the expression of PP2Ab in Jurkat cells (Supplementary Figure 2B). Confocal microscopy suggested that PP2Ab was translocated from the plasma membrane to the cytoplasm after aloperine treatment (Supplementary Figure 2C). In agreement with these results, we found that DSS decreased PP2A expression in the colon of mice, while aloperine markedly increased PP2A expression in the colons of mice from the Alo + DSS and DSS + Alo groups as compared to the DSS group (Figure 5(c)).

**Figure 6:**
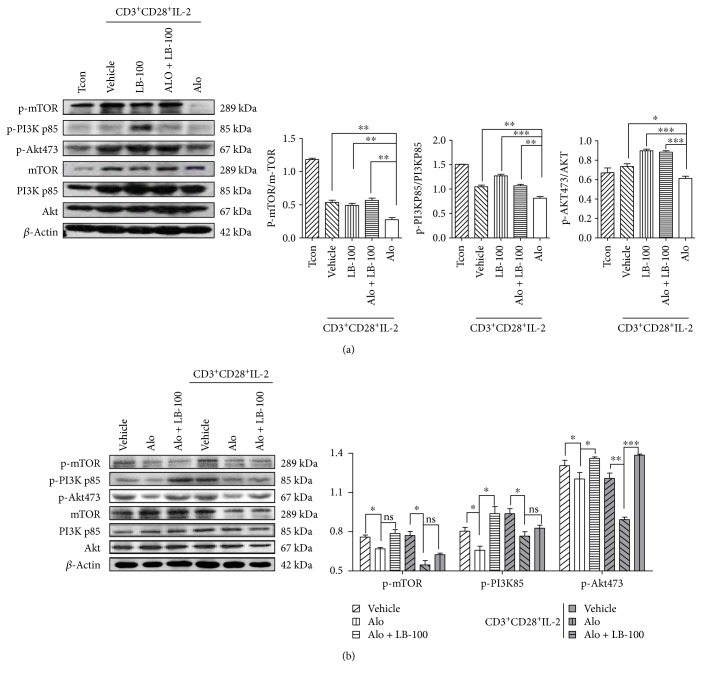
PP2A is involved in the aloperine-induced suppression of PI3K/Akt/mTOR signaling. (a) Western blotting analysis of total and phosphorylated PI3K/Akt/mTOR in conventional and activated T-cell after 24 h treatment with aloperine (0.25 mM) and LB-100 (4 *μ*M). (b) Western blotting analysis of total and phosphorylated PI3K/Akt/mTOR in activated and nonactivated human splenic mononuclear cells after 24 h treatment with aloperine (0.25 mM) and LB-100 (4 *μ*M). The data are shown as the mean ± SEM from three independent experiments. ^∗^*P* < 0.05, ^∗∗^*P* < 0.01, and ^∗∗∗^*P* < 0.001. ns: not significant.

We cultured the Jurkat cells with aloperine (0.5 mM) alone or in the presence of the PP2A inhibitor LB-100 (4 *μ*M) for 24 h. LB-100 reversed aloperine-induced suppression of PI3K/Akt/mTOR signaling (Figure 5(a)). In addition, aloperine suppressed PI3K/Akt/mTOR signaling in mouse naïve T-cells (Figure 6(a)). No difference was observed between the active and inactive human splenic mononuclear cells, suggesting that the human donor cells had been activated (Figure 6(b)). These data suggested that PP2A is involved in the regulatory effect of aloperine on PI3K/Akt/mTOR signaling.

### 3.7. PP2A Plays a Role in Aloperine-Induced Inhibition of T-Cell and Lymphocyte Proliferation and Promotion the Expression of Foxp3

**Figure 7:**
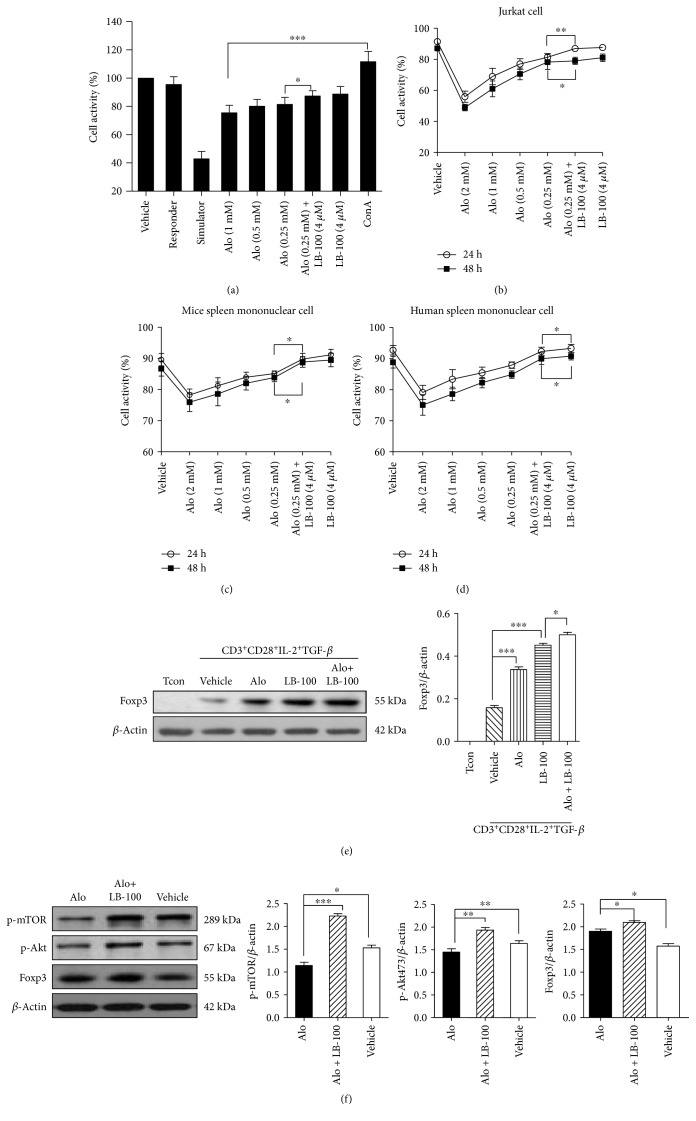
PP2A plays a role in aloperine-induced inhibition of T-cell and lymphocyte proliferation and promotion of the expression of Foxp3. (a) Cell viability was assessed in the mixed lymphocyte reaction (MLR) of a mouse by CCK-8 assay. (b, c, and d) Cell viability of Jurkat cells, mouse splenic mononuclear cells, and human splenic mononuclear cells after 24 or 48 h of aloperine and LB-100 treatments. (e) Western blotting analysis of Foxp3 in mouse T-cells following 72 h treatment with aloperine (0.25 mM) and LB-100 (4 *μ*M). (f) Western blotting analysis of p-mTOR, p-Akt, and Foxp3 in mouse Tregs following 24 h treatment with aloperine (0.25 mM) and LB-100 (4 *μ*m). The data are shown as mean ± SEM from three independent experiments. ^∗^*P* < 0.05, ^∗∗^*P* < 0.01, and ^∗∗∗^*P* < 0.001.

The mixed lymphocyte reaction (MLR) showed that the alloreactivity-induced proliferation was inhibited by high concentrations of aloperine, while LB-100 reverse this effect (Figure 7(a)). In addition, the CCK-8 assay showed that aloperine decreased the viability of Jurkat and splenic mononuclear cells in both mice and humans (Figures 7(b), 7(c), and 7(d)). LB-100 treatment alleviated the ability of aloperine to inhibit Jurkat cell apoptosis and exhibited a similar effect on primary cells. Surprisingly, both aloperine and LB-100 increased the Foxp3 levels in mouse naïve T-cells and Tregs, compared with aloperine alone (Figures 7(e) and 7(f)).

## 4. Discussion

In the present study, aloperine treatment improved colitis induced by DSS based on body weight, DAI, colon length, and SI. Aloperine also attenuated the inflammation of colitis in mouse based on the pathological score and MPO, which is a specific marker of neutrophil infiltration that is often used to assess disease activity in colitis [30]. Aloperine suppresses PI3K/Akt/mTOR signaling by promoting Foxp3^+^ Tregs, reducing T-cell activation, and inducing T-cell apoptosis (through increased Bax expression and decreased Bcl-2 and cleaved caspase-3 expression). Tregs inhibit the inflammatory responses in the intestinal mucosa and maintain the immune balance [4]. Moreover, aloperine can also promote the activation of Tregs under normal physiological conditions.

Aloperine may inhibit the excessive immune response by inhibiting the T-cell proliferation and modulating the balance between proinflammatory and anti-inflammatory factors in the intestinal mucosa, as previously observed. Indeed, Yang et al. [31] showed that aloperine decreases inflammatory pain in mice through the suppression of proinflammatory cytokines. This improved anti-inflammatory balance by aloperine could be a putative mechanism underlying aloperine-mediated protection against colitis. Taken together, we deduced that the most critical effect of aloperine treatment in colitis was blocking the CD44^high^CD62L^low^ effector/memory cells and regulating the Treg functions. No differences were observed between the two treatment groups in the proportions of Tregs and effector/memory cells, which indicated that the effects of simultaneous or subsequent aloperine treatments on T-cell differentiation were similar. The T-cells can be divided into two major subsets: naïve and activated T-cells, which include the effector and memory T-cells [32]. The naïve cells are characterized by the CD44^low^CD62L^high^ phenotype and become effector T-cells (CD44^high^CD62L^low^) when stimulated by antigens [33]. Aloperine suppressed the production of proinflammatory cytokine IL-17A and declined the IL17A/Foxp3 ratio and effector T-cell proliferation, upregulated the expansion of memory T-cell, and promoted the proliferation of Tregs, thereby inhibiting excessive immune response in the intestine. Our results confirmed that aloperine regulated the immune balance by controlling the proportion and functions of Tregs and relative cytokines, IL-10 and TGF-*β*.

Aloperine induces G2/M phase cell cycle arrest and apoptosis in human colon cancer cells [10]. PP2A is considered a proapoptotic factor that inhibits cell mitosis through phosphorylation during the cell cycle, mostly during the G2/M phases [34]. Aloperine regulates the expression and location of PP2A and T-cell apoptosis in colitis through increased Bax expression and decreased Bcl-2 and cleaved caspase-3 expression. Indeed, our data indicated that PP2A might be vital in mediating the protective effects of aloperine. This phenomenon indicated that PP2A is differentially regulated with respect to Treg frequency and function. Therefore, aloperine modulates the intestinal immune system through PI3K/Akt/mTOR pathway and PP2A is a vital regulatory protein. We postulated that aloperine induces the apoptosis of T-cells and Jurkat cells and inhibits their proliferation. The inhibition of PP2A abolished the suppressive effect, which might be ascribed as the underlying mechanism for aloperine-attenuated murine colitis.

The annihilation of PP2A might compromise Treg functions. PP2A knockout mice were confirmed to be with lymphoproliferative and autoimmune disorders, as shown by greater Treg cell frequency in the spleen and lymph nodes [35]. In addition, PP2A has been shown to enhance the inflammatory effects of a number of compounds [36, 37] and PP2A activation attenuates inflammation in animal models [38, 39]. These results were consistent with the present study, wherein we showed that PP2A expression declined in colitis and LB-100 increased the expression of Foxp3 in mouse naïve T-cells and Tregs. Conversely, a previous study demonstrated that mice fed DSS for 3–12 weeks exhibited PP2A methylation, which might cause inflammation [40]. The change in methylation may be detected after an extended period, and this discrepancy might be due to the different durations of treatments in animal models as our experiment lasted only 2 weeks and represented an acute inflammation model.

We demonstrated that aloperine suppressed PI3K/AkT/mTOR signaling, a pathway that positively regulates T-cell functions [9, 41, 42]. This phenomenon may be a pivotal mechanism underlying the aloperine-mediated therapeutic effect on colitis. We used *in vivo* and *in vitro* experiments to confirm that aloperine suppressed PI3K/Akt/mTOR signaling and promoted Treg differentiation and activation in mice; similar results were obtained in human splenic cells. The inhibition of the PI3K/Akt/mTOR signaling has been shown to have benefits in a number of inflammatory condition [43–45]. The PI3K/Akt/mTOR signaling pathway negatively modulates the functions of Tregs; the PI3K activity is most abundant among proinflammatory cells within the stroma [46]. PP2A might inactivate PI3K and abrogate the suppressive capacity of Treg cells. The present study suggests that PP2A is upstream of PI3K/Akt/mTOR, but the mechanism which is the relationship between PI3K/Akt/mTOR pathway and PP2A is not yet elucidated. Furthermore, PP2A is a signaling phosphatase that has been implicated in the regulation of Akt activities [47, 48]. PP2A may directly interact with Akt that mediates the suppressive effect of aloperine on PI3K signaling. In order to determine whether PP2A is a direct target of aloperine, further studies are needed. In addition, the relationships among Tregs, PI3K/Akt/mTOR pathway, and PP2A need to be further studied.

The literature proposes two routes for aloperine administration: intragastric administration and intraperitoneal injection [11, 12]. In the first stage of the study, we found that intragastric administration of aloperine could promote the expression of Treg in DSS-induced colitis mouse model. Therefore, in the second stage, the other route was tried to verify the effect of aloperine on the expression of Treg in the *Foxp3*^GFP^ mice. We agree that we could have used the same route in both parts of the experiment, but a confirmation that both routes worked was wanted. As for the dose, our previous pilot experiments (data not shown) suggested that a 20 mg/kg dose was enough when the intraperitoneal route was used. Indeed, when using the intragastric route, it is probable that not all aloperine is absorbed by the intestine. This will have to be explored in future studies.

## 5. Conclusions

Aloperine regulates the inflammatory responses in colitis by inhibiting the PI3K/Akt/mTOR signaling in a PP2A-dependent manner.

## Supplementary Material

Supplementary Figure 1. Schematic illustration of the C57BL/6 mice randomly divided into five groups. i.g.: intragastric administration; DSS: Dextran sodium sulfate. Supplementary Figure 2. Effects of aloperine on PI3K/Akt/mTOR signaling and PP2Ab in Jurkat cells. (A) Western blot analysis of PI3K/Akt/mTOR signaling in Jurkat cells after 24 h treatment with different concentrations (0.25, 0.5, or 1 mM). (B) Western blot analysis of PHLPP1, PML, and PP2Ab after 24 h treatment with different concentrations (0.25, 0.5, or 1 mM). (C) Immunofluorescent staining of PP2Ab in Jurkat cells after treatment with 0.5 mM aloperine for 48 h.

## Figures and Tables

**Table 1:** Primers used for real-time PCR.

Gene	Primer (5′ → 3′)
*IL-17A*	F: CTCAGACTACCTCAACCGT R: CTTTCCCTCCGCATTGACA
*Foxp3*	F: GCC CAC CAG TAC AGC TGG A R: ACT CTG CCT TCA GAC GAG ACT TG
*IL-10*	F: CCC TTT GCT ATG GTG TCC TT R: TGG TTT CTC TTC CCA AGA CC
*TGF-β*	F: CAA CAA TTCCTG GCG TTA CCT TGG R: GAA AGC CCT GTA TTC CGT CTC CTT
*β-Actin*	F: TCA TCA CTA TTG GCA ACG AGC R: AAC AGT CCG CCT AGA AGC AC

IL-17A: interleukin-17A; FOXP3: forkhead box P3; IL-10: interleukin-10; TGF-*β*: tumor growth factor-*β*; F: forward; R: reverse.
