# Supplementary material for: Aloperine Protects Mice against DSS-Induced Colitis by PP2A-Mediated PI3K/Akt/mTOR Signaling Suppression
Source: Mediators Inflamm. 2017 Sep 19;2017:5706152. doi: 10.1155/2017/5706152 (PMC5625759; doi:10.1155/2017/5706152)

Supplementary Figure 1. Schematic illustration of the C57BL/6 mice randomly divided into five groups. i.g.: intragastric administration; DSS: Dextran sodium sulfate.

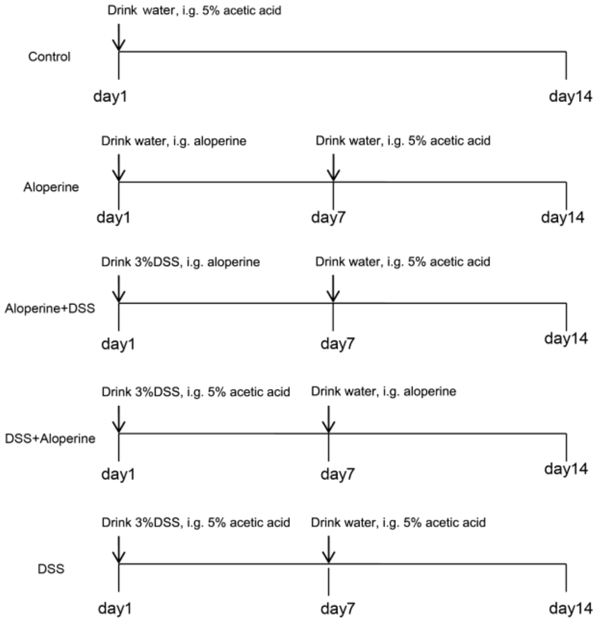

Supplementary Figure 2. Effects of aloperine on PI3K/Akt/mTOR signaling and PP2Ab in Jurkat cells. (**A**) Western blot analysis of PI3K/Akt/mTOR signaling in Jurkat cells after 24 h treatment with different concentrations (0.25, 0.5, or 1 mM). **(B)** Western blot analysis of PHLPP1, PML, and PP2Ab after 24 h treatment with different concentrations (0.25, 0.5, or 1 mM). **(C)** Immunofluorescent staining of PP2Ab in Jurkat cells after treatment with 0.5 mM aloperine for 48 h.

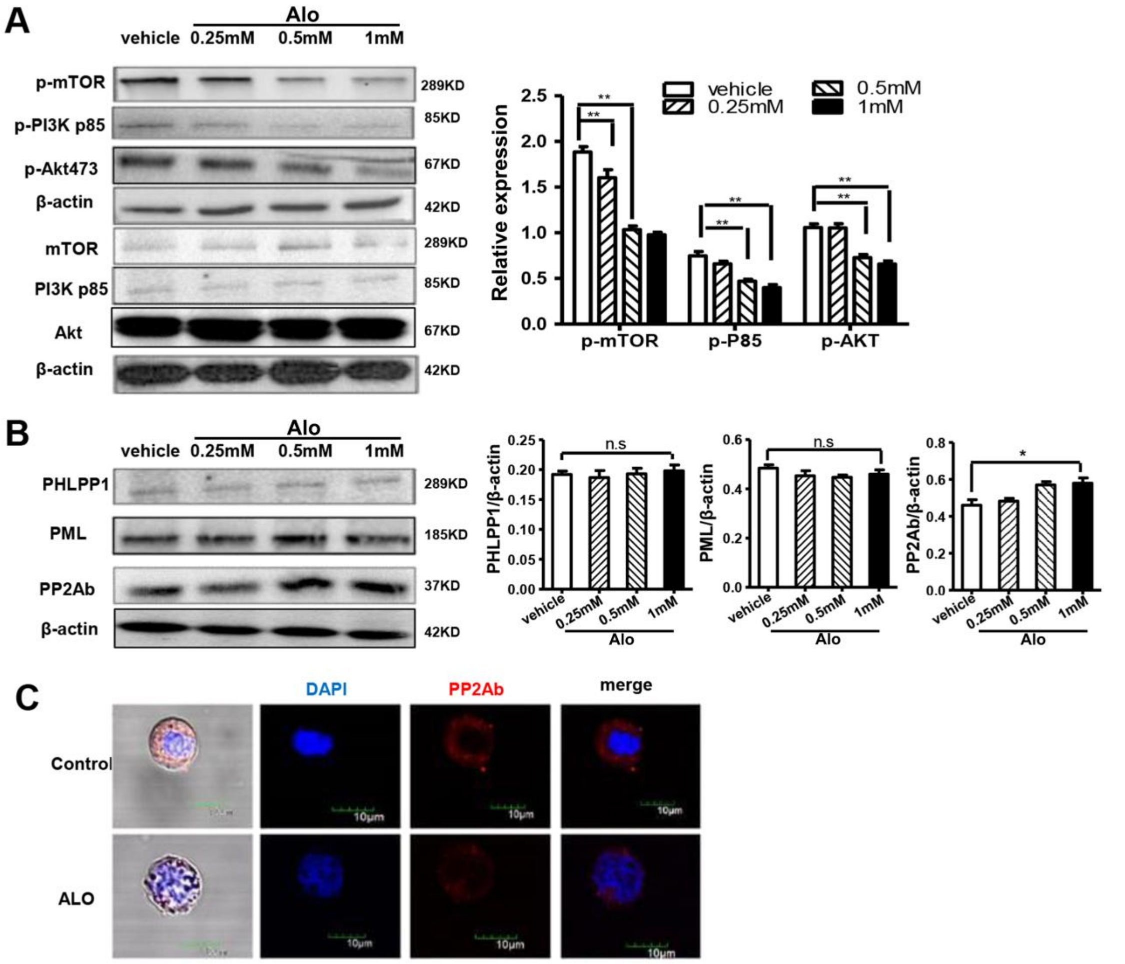

Supplement: Supplementary file 1 — Supplementary Figure 1. Schematic illustration of the C57BL/6 mice randomly divided into five groups. i.g.: intragastric administration; DSS: Dextran sodium sulfate. Supplementary Figure 2. Effects of aloperine on PI3K/Akt/mTOR signaling and PP2Ab in Jurkat cells. (A) Western blot analysis of PI3K/Akt/mTOR signaling in Jurkat cells after 24 h treatment with different concentrations (0.25, 0.5, or 1 mM). (B) Western blot analysis of PHLPP1, PML, and PP2Ab after 24 h treatment with different concentrations (0.25, 0.5, or 1 mM). (C) Immunofluorescent staining of PP2Ab in Jurkat cells after treatment with 0.5 mM aloperine for 48 h. [file 5706152.f1.pdf]
